# Salicylic Acid Attenuates Gentamicin-Induced Nephrotoxicity in Rats

**DOI:** 10.1100/2012/390613

**Published:** 2012-04-30

**Authors:** Pavle Randjelovic, Slavimir Veljkovic, Nenad Stojiljkovic, Ljubinka Jankovic-Velickovic, Dusan Sokolovic, Milan Stoiljkovic, Ivan Ilic

**Affiliations:** ^1^Department of Physiology, Faculty of Medicine, University of Nis, 18000 Nis, Serbia; ^2^Department of Pathology, Faculty of Medicine, University of Nis, 18000 Nis, Serbia; ^3^Department of Biochemistry, Faculty of Medicine, University of Nis, 18000 Nis, Serbia; ^4^Department of Pharmacology, Faculty of Medicine, University of Nis, 18000 Nis, Serbia

## Abstract

Gentamicin (GM) is a widely used antibiotic against serious and life-threatening infections, but its usefulness is limited by the development of nephrotoxicity. The present study was designed to determine the protective effect of salicylic acid (SA) in gentamicin-induced nephrotoxicity in rats. Quantitative evaluation of gentamicin-induced structural alterations and degree of functional alterations in the kidneys were performed by histopathological and biochemical analyses in order to determine potential beneficial effects of SA coadministration with gentamicin. Gentamicin was observed to cause a severe nephrotoxicity which was evidenced by an elevation of serum urea and creatinine levels. The significant increases in malondialdehyde (MDA) levels and protein carbonyl groups indicated that GM-induced tissue injury was mediated through oxidative reactions. On the other hand, simultaneous SA administration protected kidney tissue against the oxidative damage and the nephrotoxic effect caused by GM treatment. Exposure to GM caused necrosis of tubular epithelial cells. Necrosis of tubules was found to be prevented by SA pretreatment. The results from our study indicate that SA supplement attenuates oxidative-stress associated renal injury by reducing oxygen free radicals and lipid peroxidation in gentamicin-treated rats.

## 1. Introduction

Gentamicin (GM) is commonly applied in human clinical practices for treatment of life-threatening gram-negative infections [1, 2]. However, the usefulness of GM is limited by the development of nephrotoxicity. In some cases, this side effect is so severe that the use of the drug must be discontinued. In spite of the introduction of newer and less toxic antibiotics, GM is still used clinically because of its rapid bactericidal action, broad-spectrum activity, chemical stability, and low cost [3, 4]. GM-induced nephrotoxicity is characterized by direct tubular necrosis, without morphological changes in glomerular structures [1]. The mechanisms involved in GM-induced cell injury are not clearly understood. However, several studies demonstrated that reactive oxygen species (ROS) may be important mediators in GM-induced nephrotoxicity [5]. Abnormal production of ROS directly damages some macromolecules and induces cellular injury and necrosis via several mechanisms including peroxidation of membrane lipids, protein denaturation, and DNA damage [6, 7]. Accordingly, the administration of several compounds with antioxidant activity has been successfully used to prevent or ameliorate GM-induced nephrotoxicity [8, 9].

In the past few years, much interest has been laid on the role of naturally occurring dietary substances for the control and management of various chronic diseases, one such compound salicylic acid (SA) has been used since ancient times to provide pain relief and treat inflammatory conditions. Salicylic acid is a phenolic compound present in plants, where it plays a central role in the development of local and systemic resistance to pathogen infection [10, 11]. Humans and animals obtain SA mainly from daily foods, fruits, and vegetables. Increasing evidence demonstrates that applied SA can counteract oxidative damage induced by adverse conditions in animals [12, 13], though the mechanisms underlying these effects remain unclear. It has been reported that SA comprise free radical-scavenging and iron chelation properties [14]. SA can affect the activation of transcription factors, in particular nuclear factor kappa B (NF-*κ*B), thereby intervening in apoptotic pathways [15]. It is also a hydroxyl radical scavenger in both experimental animals and humans who are experiencing oxidative stress [16, 17].

The aim of the present study was therefore to investigate whether SA treatment prevents GM-induced nephrotoxicity. For this purpose, we have examined histopathological effects of GM and possible protective effect of SA on tissue damage of rat kidney. We have also examined tissue malondialdehyde (MDA) and protein carbonyl levels in order to evaluate lipid and protein peroxidation, and serum urea and creatinine levels in order to evaluate renal function.

## 2. Material and Methods

Thirty-two healthy adult female Wistar albino rats weighing 250–300 g were randomly selected for this study. The animals were placed in a temperature- (21 ± 2°C) and humidity-controlled room with 12-hour light-dark cycles and fed standard pellet chow and water ad libitum. All experimental procedures were conducted in accord with the principles for the care and use of laboratory animals in research and approved by the local ethics committee. All efforts were made to minimize animal suffering and reduce the number of animals used.

### 2.1. Experimental Protocol

 After a quarantine period of 7 days, 32 rats were randomly divided into four groups, each consisting of 8 animals. Group I was used as control and received 1 mL of saline intraperitoneally (i.p.) per day. Group II received only salicylic acid in single dose of 100 mg/kg i.p. daily. Group III received gentamicin (Galenika AD, Belgrade, Serbia) on a daily basis in a single dose of 100 mg/kg by i.p. injection. Group IV was given salicylic acid (Sigma, St. Louis, MO, USA) in a single i.p. dose of 100 mg/kg along with the same dose of gentamicin as the group II each day throughout the experiment. All groups were treated over a period of 8 consecutive days. Twenty-four hours after the administration of last doses of GM and SA, on 9th day, rats were anesthetized by intraperitoneal injection of ketamine (Ketamidor 10%, Richter Pharma AG, Wels, Austria) and sacrificed. Renal cortical tissues were separated into two parts for biochemical analysis and light microscopic examination. Blood samples were also taken by cardiac puncture to assess the serum levels of urea and creatinine.

### 2.2. Biochemical Analysis

Serum urea and creatinine levels were determined with an automatic biochemical analyzer (A25 Biosystems, Barcelona, Spain).

For estimation of oxidative stress the kidney tissue was cut in small pieces and homogenized in ice-cold water, by using a homogenizer (IKA Works de Brasil Ltda Taquara, RJ 22713–00). The homogenates (10% w/v) were centrifuged at 1500 × g for 10 min. at 4°C.

### 2.3. Determination of Proteins

Proteins were determined according to Lowry's method [18] using bovine serum albumin as standard.

### 2.4. Determination of MDA

The intensity of lipid peroxidation in the kidney tissue was spectrophotometrically measured, based on the thiobarbituric (TBA) response products [19]. Homogenate absorption was measured at 532 nm. Malondialdehyde-(MDA-)lipid peroxidation end product, concentration was expressed per mg/protein, using the molecular extinction coefficient of MDA (1.56 × 10^-5 ^mol cm^−1^).

### 2.5. Determination of Protein Oxidation

Carbonyl group concentration, as the level of oxidative modified proteins, was determined spectrophotometrically [20] using 2.4 dinitrophenylhydrazine, a traditional carbonyl reagent. Reactive carbonyl derivatives were calculated using the DPNH molar extinction coefficient at 370 nm (22 × 10^3^ L/mol/cm) and expressed in *μ*mol/g of protein.

### 2.6. Histopathological Examinations

Histopathological evaluation was made in kidney tissues. Kidneys were dissected immediately and preserved in 10% buffered formaldehyde for further histopathological examinations. Tissue samples were embedded in paraffin and 5-6 *μ*m sections were cut using a rotary microtome and stained with hematoxylin and eosin (H&E). A minimum of 8 fields for each kidney section were examined and assigned for severity of changes by an observer blinded to the treatments of the animals. All sections were examined with a Leica DMR (Leica Microsystems AG, Wetzlar, Germany) light microscope. To evaluate the level of damages, indexes such as tubular degeneration, tubular necrosis, mononuclear cell infiltration, and hyaline casts were scored numerically. The evaluation criteria were as follows: 0 for no detectable lesion, 1 for mild changes, 2 for moderate changes, and 3 for severe changes.

### 2.7. Statistical Analysis

Results were expressed as the mean ± SD. Statistical significant difference was determined by one-way analysis of variance (ANOVA) followed by Tukey's post hoc test for multiple comparison (Graphpad Prism version 5.03, San Diego, CA, USA). Probability values (*P*) less than 0.05 were considered to be statistically significant.

## 3. Results

### 3.1. Effect of SA on Serum Creatinine and Urea Levels in Gentamicin-Treated Rats

GM treatment for eight days resulted in significant increase in serum creatinine and urea compared to control rats (Table 1). However, elevations in the serum creatinine and blood urea were significantly (*P* < 0.001) attenuated by SA pretreatments, indicating reduction in GM-induced nephrotoxicity (*P* < 0.01, resp.). SA treatment alone did not change the renal function tests, when compared to control values (Table 1).

### 3.2. Effect of SA on Renal Oxidative Stress in Gentamicin-Treated Rats

**Figure 1:**
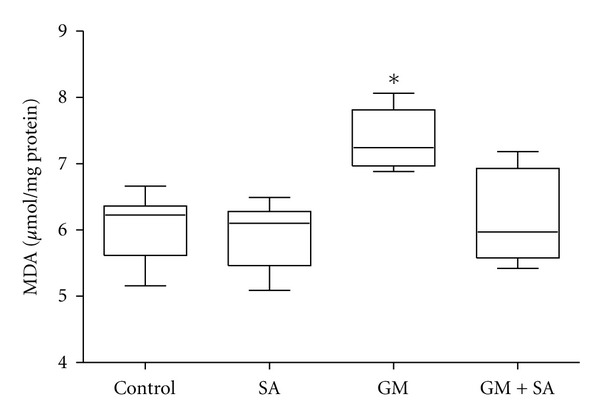
Effect of gentamicin (GM), salicylic acid (SA) and their combination on malondialdehyde (MDA) levels in kidney tissues of rats. Values are means ± SD. *P < 0.001 versus Control, SA and GM.

Tissue MDA was significantly increased (7.36 ± 0.45 *μ*mol/mg) in the GM-treated renal injury group, when compared to the control group (6.07 ± 0.54 *μ*mol/mg; *P* < 0.001). The increases induced by GM were completely prevented by SA administrations (GM + SA group). The MDA contents were found similar in the control and SA groups (Figure 1).

**Figure 2:**
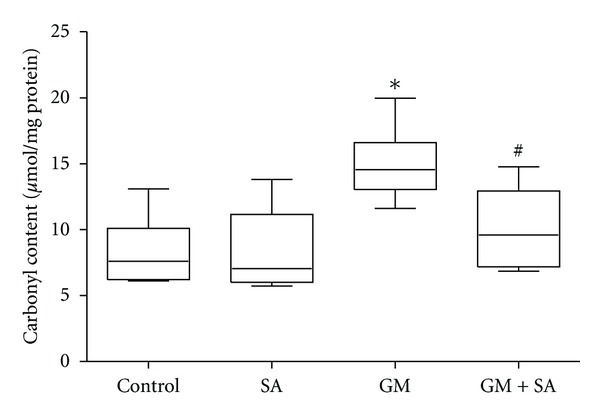
Effect of gentamicin (GM), salicylic acid (SA), and their combination on protein carbonyl content in kidney tissues of rats. Values are means ± SD. *P < 0.001 versus control and SA; ^#^ *P* < 0.01 versus GM.

GM treatment induced a significant increase in the protein carbonyl content in renal tissue compared to the control group (Figure 2). Renal protein carbonyl content of the GM group (14.99 ± 2.58 *μ*mol/mg) was attenuated by treatment with SA (10.12 ± 3.27 *μ*mol/mg; *P* < 0.01). SA treatment alone did not cause a significant effect on protein carbonyl content of the control group (Figure 2).

### 3.3. Histopathological Analysis

**Figure 3:**
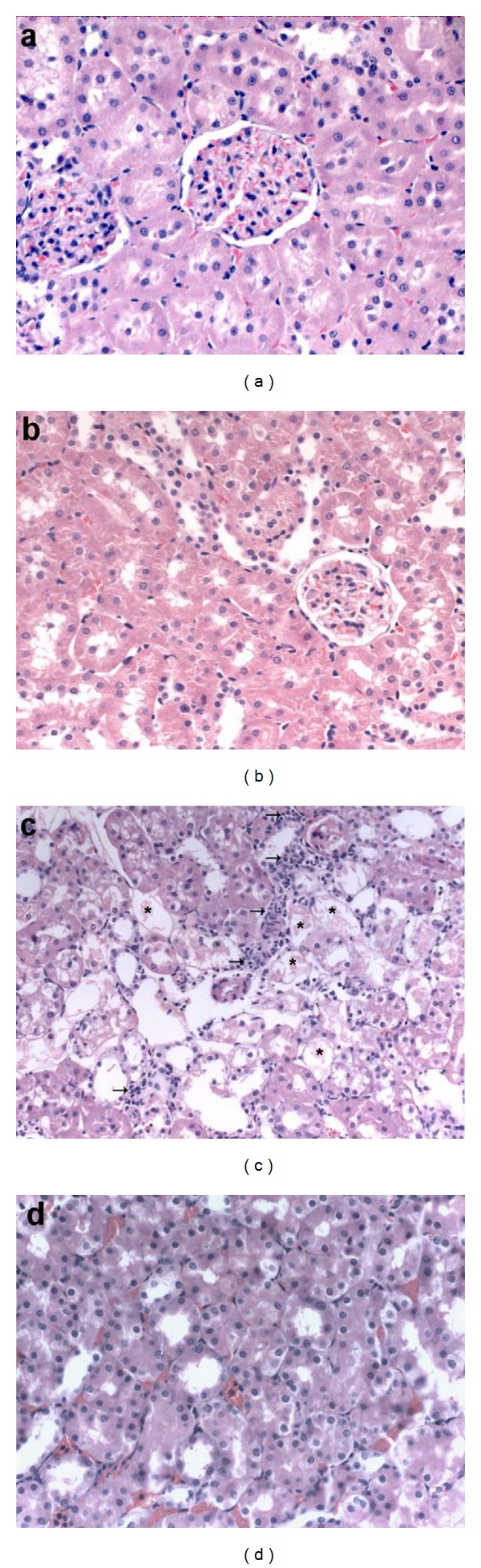
Photomicrograph of rat kidney section. (a and b) Normal histology of kidney tissue in control and SA-treated rats (H&E ×200). (c) Marked tubular necrosis (asterisk) and massive mononuclear cell infiltration (arrow) in cortex of rats in GM-group (H&E ×200). (d) Section from rat treated with gentamicin (100 mg/kg) plus salicylic acid (100 mg/kg) reveal almost complete prevention of histopathological alterations (H&E ×200).

The histopathological changes in kidneys in all groups are summarized in Table 2. Light-microscopic examination of kidneys from control and SA-treated rats showed no structural alterations in renal tissues (Figures 3(a) and 3(b)). Massive and diffuse cell necrosis was observed in the proximal tubules of kidneys from rats injected with gentamicin. In addition, the lumens of these tubules were filled with degenerate and desquamated epithelial cells and hyaline casts. Severe inflammatory infiltrate in the form of mononuclear cells were observed in the renal sections of this group (Figure 3(c)). Kidney specimens from rats treated with GM and SA revealed significant improvement in glomeruli and renal tubules, evidenced by preservation of tubular histology compared with the GM-treated group (Figure 3(d)).

## 4. Discussion

Aminoglycoside antibiotic GM is commonly used for the treatment of severe gram-negative bacterial infections [21]. However, nephrotoxicity is a major complication of GM administration. Thus amelioration of nephrotoxicity would enhance its clinical use. Several approaches involving the use of chemical compounds have been used to reduce GM nephrotoxicity [7, 22]. Phenolic compounds from dietary plants are known to be good scavengers of reactive oxygen species. In the past few decades, a considerable and consistent amount of evidence has demonstrated that SA has antioxidant properties [23, 24], though the mechanisms underlying these effects remain unclear. Firstly, it has been reported that salicylates comprise free radical-scavenging and iron chelation properties [14]. Also, it has been demonstrated that salicylate effectively protects against gentamicin-induced hearing loss in guinea pigs [25]. Thus in the present study, we assessed whether the nephrotoxic effects caused by acute administration of GM could be prevented or ameliorated by treatment with SA, a herbal compound which possesses a strong antioxidant property [23]. Several dosage schemes have been reported for GM administration and an intraperitoneal (i.p.) dose of 100 mg/kg body weight, for 8 days, was used which is a dosage scheme reported to cause significant nephrotoxicity in rats [26].

The results of this study show that GM administration to rats produced a typical pattern of nephrotoxicity which was manifested by marked increase in serum creatinine and urea levels. On the other hand, SA administration showed a significant decrease in the levels of serum creatinine and urea. The curative effect of SA on the kidney markers can be attributed to its antioxidant property as it has been found that ROS may be involved in the impairment of glomerular filtration rate (GRF) [27]. Low or moderate production of ROS plays a physiological role in several redox-responsive signaling pathways, for example, in defense against environmental pathogens, regulation of vascular tone by nitric oxide (NO), regulation of cell adhesion, and apoptosis [28]. Nevertheless, when these reactive species are sustainably produced in overwhelming amounts, they may initiate a wide range of pro-oxidant reactions that result in damage of cellular macromolecules, including lipid peroxidation, protein nitration, and oxidation [28]. Formation of ROS has been shown to increase with GM treatment [22]. This evolution of ROS would stimulate the activation or expression of proinflammatory mediators, including NF-*κ*B, leukocyte adhesion molecules, and mitogen-activated protein kinases (MAPKs) [29], which contribute to progressive kidney damage induced by GM. Recent studies have shown that redox-sensitive transcription factors, MAPK and NF-kB, are involved in nephrotoxicity caused by GM [30, 31]. NF-*κ*B is a highly conserved family of transcription factors that has a critical role in mediating inflammation, apoptosis, and growth in chronic disease [32]. Activation of NF-*κ*B, in response to oxidative stress might play a role in GM-induced nephrotoxicity by inducing synthesis of inflammatory substances (cytokines, growth factors, and adhesion molecules) that provoke kidney damage [33]. Thus, blockade of NF-*κ*B will be an effective approach for the treatment of nephrotoxicity.

SA has been shown to block NF-kB-mediated gene expression at suprapharmacological concentrations [34]. In LPS-stimulated cells, SA blocked the LPS-induced phosphorylation and proteolysis of inhibitors of kB (I*κ*B), which suggests that the inhibition of NF-*κ*B was mediated through the inactivation of the classical signaling pathway [34]. The finding that NF-*κ*B activity is inhibited by salicylates indicates that their anti-inflammatory activity is partially related to the inhibition of this transcription factor.

As expected, MDA and carbonyl group levels increased significantly in the kidney of rats exposed to GM compared to the control group. These oxidative stress-related alterations, which are in agreement with previous reports [35], were attenuated by SA administration. A plausible justification for this protection conferred by SA is its potent scavenging effect on hydroxyl radical (HO·). Among ROS, HO· is thought to be the most damaging species and the one mainly responsible for lipid and protein oxidation [36].

These findings correlated well with the histological examination, which revealed tubular necrosis especially in the renal cortex (Table 2). The kidneys of the control group showed normal histological features (Figure 3(a)), but the GM-treated group revealed more extensive and marked tubular necrosis. There were leukocytic infiltrations considered, as a prominent response of the body tissue facing any injurious impacts (Figure 3(c)). These modifications could be due to the accumulation of free radicals resulting from an increased lipid peroxidation in the renal tissues of the GM-treated group. Renal lesions were also characterized by vascular congestion as well as tubular obstruction. Similar changes were also reported by Kumar et al. [37] and Stojiljkovic et al. [38] who demonstrated structural changes in renal tissue of GM-treated animals and its reversal by various agents. Glomerular and tubular epithelial changes were considerably mild in the group treated with both GM + SA (Figure 3(d)), thus showing curative effect of SA against GM-induced tissue damage.

Besides their direct damaging effects on tissues, free radicals seem to trigger the accumulation of leukocytes in the tissue involved, and thus cause tissue injury also indirectly through activated neutrophils. It has been shown that activated neutrophils secrete enzymes (e.g., myeloperoxidase, elastase, and proteases) and liberate oxygen radicals [39]. Increasing evidence suggests that mesangial cells and neutrophils release chemotactic substances (e.g., interleukin 8), which further promote neutrophil migration to the kidney, activate neutrophils, and increase glomerular injury [40]. These results suggest that neutrophils play an important role in mediating tissue injury with subsequent renal failure [41]. Oxidative stress and inflammation are inextricably linked as one begins and amplifies the other. In this context, oxidative stress invariably recruits inflammation via activation of NF-*κ*B, which is the general transcription factor for various proinflammatory cytokines, chemokines, and adhesion molecules. Production of these mediators promotes leukocyte/macrophage adhesion, activation, infiltration, and ROS production. The latter, in turn, accentuate the inciting oxidative stress. Conversely, release of ROS, reactive chlorine, nitrogen, and other species by activated leukocytes and macrophages in the course of the primary inflammation results in oxidative stress [42].

## 5. Conclusion

The results of this study confirm the earlier reports that GM-treated rats show accelerated lipid and protein oxidation in the renal tissue, as reflected by an increase in MDA and protein carbonyl groups. Pretreatment with SA afforded significant protection against nephrotoxicity induced by GM treatment. The beneficial effect of SA in GM toxicity implies the involvement of free radicals in the renal damage. According to our biochemical findings, which were supported by histopathological evidence, administration of SA abolished nephrotoxic effects of GM. These findings indicate that SA supplementation may reduce GM-induced renal injury. We propose that salicylic acid modulates oxidative stress and associated potentially proinflammatory activity in the kidney. This may be via mechanisms linked to redox signaling, through an effective inhibition of proinflammatory factors, scavenging of ROS, and inhibition of NF-*κ*B.

## Figures and Tables

**Table 1:** Effects of salicylic acid on gentamicin-induced renal dysfunction as measured by levels of serum urea and creatinine.

Parameters	Control	SA	GM	GM + SA
Urea (mmol/L)	5.60 ± 0.91*	5.20 ± 0.66*	18.91 ± 2.86	9.53 ± 1.43^#^
Creatinine (*μ*mol/L)	48.63 ± 2.34^#^	51.75 ± 8.26^#^	71.71 ± 9.43	55.88 ± 8.82^#^

Data are presented as mean ± SD.

*P < 0.001 versus GM, GM + SA.

^#^ *P* < 0.001 versus GM.

**Table 2:** Grading of histopathological changes in the kidney sections.

Histopathological changes	Control	SA	GM	GM + SA
Mononuclear cell infiltration	—	—	+++	+
Tubular degeneration	—	—	+++	+
Tubular necrosis	—	—	++	—
Hyaline casts in tubular lumen	—	—	+	—

Scoring was done as follows: none (—), mild (+), moderate (++), and severe (+++).
